# Singular Value Decomposition of the Radial Distribution Function for Hard Sphere and Square Well Potentials

**DOI:** 10.1371/journal.pone.0075792

**Published:** 2013-10-15

**Authors:** Travis Hoppe

**Affiliations:** National Institutes of Health, National Institute of Diabetes and Digestive and Kidney Diseases, Bethesda, Maryland, United States of America; University of Calgary, Canada

## Abstract

We compute the singular value decomposition of the radial distribution function 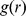 for hard sphere, and square well solutions. We find that 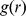 decomposes into a small set of basis vectors allowing for an extremely accurate representation at all interpolated densities and potential strengths. In addition, we find that the coefficient vectors describing the magnitude of each basis vector are well described by a low-order polynomial. We provide a program to calculate 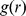 in this compact representation for the investigated parameter range.

## Introduction

To date, there have been many numerical calculations of the radial distribution function (RDF) and equations of state for discrete potentials [1]–[11]. Analytical theories arising from closures of the Ornstein-Zernike equations, such as the Percus Yevick or Hypernetted-chain, currently validate their predictions on a set of fixed simulation data rather than a continuous range of parameters. In this work, we present a reduced representation for a continuous range of hard sphere and square well parameters that reproduces simulation data to extremely high accuracy.

Since the Fourier transform of 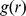 results in the experimentally measurable structure factor, prediction of the RDF is one of the core goals of liquid state theories. This work was motivated by the lack of an accurate tabulation of 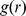 for various idealized fluids. Such a tabulation would hold value for validating both theoretical predictions and modeling empirical results. There are numerous theories that rely on “apparent” hard-sphere volumes or fitted square well parameters to predict observed properties [12]–[16]. With a smooth interpolation, not only would the fits be more accurate, the sensitivity to inputs could be measured as well. In addition, modeling across a larger parameter domain would be possible with a two (or higher) dimensional parameterization. In this paper we show the results of a systematic computational study of the RDF for two well-studied potentials, the hard sphere (HS) and a square well (SW). We find that the potential in all cases can be decomposed into very few basis vectors when the density and attractive strength are varied. This allows for the RDF to be determined at all interpolated values with high accuracy.

Our approach is similar to the work of di Dio, et. al. [17] where the basis vectors, as a function of temperature, were used to model the RDF of water. Due to the complexity of the model, only five points in the parameter space were sampled. Since HS and SW potentials are less complex, we can examine several orders of magnitude more points in parameter space. This makes the singular value decomposition much more accurate and reveals several interesting phenomena.

The first is that the decomposition is very clean, in that the singular values are well separated from each other when considering variations in both density and attractive strength. This leads to a reduced representation of the RDF across the parameter range. However, variations of the SW length did not permit separation into a small number of components, consequently this parameter did not lend itself to a reduced representation.

Secondly, we note the dependence of each basis vector as a function of the system parameters was well described by a low-order polynomial. The degree of this polynomial increased linearly with the rank order of the basis vector. This means that a reconstruction of the RDF for any interpolated value of packing fraction or attractive strength can be expressed by a set of polynomial coefficients, a vector of singular values, and a small set of vectors describing the basis functions. This compact representation along with a program to reconstruct the RDF is given in the Supplementary Information.

## Methods

For a given system, we compute the radial distribution function 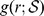 as a function of the separation *r* and a set of parameters 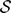. Discrete molecular dynamics (DMD) was used to simulate all systems in this study. We used a simplified set of parameters as input to an all-atom DMD program created by the Dokholyan group [18], [19]. Although originally designed to model proteins, the discrete, isotropic, pairwise additive potentials were a natural fit. For continuous potentials, the motion can only be approximately integrated over a finite time step. The event-driven nature of DMD allows for both instantaneous and exact propagation of the equations of motion. In addition, DMD allowed for rapid decorrelation of the molecular positions ensuring ergodicity and full sampling without the worry of a properly sized displacement step required for Monte Carlo simulations.

The packing fraction 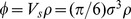 is defined by the product of the density of the spheres *ρ* and the volume occupied by a single sphere 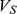. For fixed values of *N* and *φ*, a simulation box with periodic boundary conditions and length *L* was constructed such that 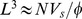. At the maximum packing fraction used and 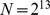 particles, this gave a box length of 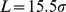. The particles were arranged by building the indices to a corresponding face-centered cubic lattice and placing particles at random until the desired density was reached. During the first phase of equilibrium, the potential (if present) was switched off to allow rapid decorrelation of the initial coordinates. During the second phase of the equilibrium, the potential was turned back on. For each phase the system equilibrated until the pressure reached a steady-state value. After equilibration, data was collected at 10^4^ equal time intervals. Since temperature is irrelevant in a HS DMD simulation, a single time step corresponded approximately 60 collisions per particle at the largest packing fractions and approximately 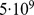 total collisions. We discretize the range 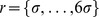 into 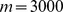 equally sized intervals, a resolution of 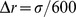. We repeat the measurement of the RDF over *k* equally distributed points in parameter space 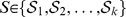.

For hard spheres the system is completely parameterized by the packing fraction 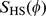. For square wells the system is parameterized by 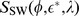, where *λ* is the well length and 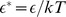 is the reduced attractive strength. With spheres of diameter *σ* the potential for a SW system is
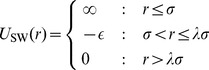
(1)where hard spheres are the limiting case of square wells as 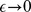. For notational convenience, we henceforth set *kT* = 1 and simply refer to 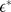 as 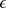.

Let 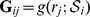 be a 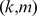 real matrix where each row represents a measurement of the RDF at a particular point in parameter space. The singular value decomposition (SVD) is
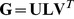
(2)The matrices 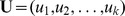 and 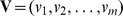 are orthogonal. Since there are more sampled intervals in 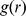 than points in parameter space, 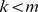, 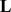 is a diagonal matrix of *k* real-valued non-negative singular values, 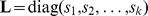 With no loss of generality, we order the singular values such that 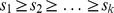.

If there is a sharp decay in the spectrum of singular values, in that 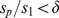 for some *p* and threshold *δ*, the original matrix 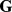 can be well approximated by the reconstruction from a partial set of the SVD
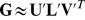
(3)where 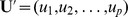 and similarly for 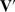 and 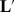. For convenience, we designate the vectors 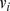 as *basis vectors* and the vectors 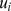 as *coefficient vectors*. While the basis vectors are a function of distance, 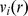, the coefficient vectors are functions of the parameters, 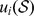. The coefficient vectors describe how each basis function contributes as a function of the system parameters. The basis vectors are equivalent to the so-called “Grund” vectors of di Dio et. al [17]. For comparisons sake, our reconstructed matrix is the transpose of Equation (3) in the referenced paper. This has the effect of switching the interpretation of the 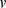 (basis) vectors with that of the 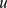 (coefficient) vectors.

The measurement of the RDF in a canonical ensemble becomes problematic in the presence of a phase separation due to finite size boundary effects. We therefore restrict the parameters to regions containing only a single liquid or vapor phase. We note that it should be possible to perform the simulation under a grand canonical ensemble. The so-called Gibbs ensemble method popularized by Panagiotopoulos [20] could provide information at the coexistence curve. For HS we examine the range from very low densities up to the freezing transition 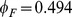, where the phase diagram becomes meta-stable [21]. For SW we restrict the domain to the exterior of the liquid-vapor coexistence curve temperatures and the freezing transition. We find good results independent of the liquidus line at the well length we considered 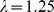.

## Results

### Hard Spheres

**Figure 1:**
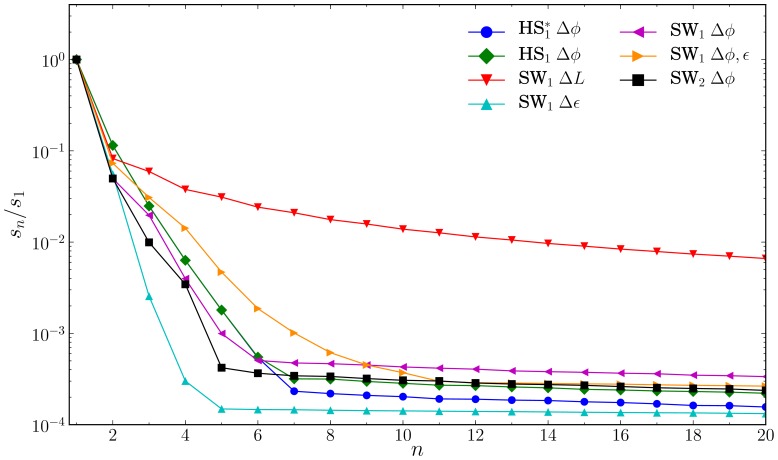
Rank sorted normalized singular value spectrum. HS, SW_1_, SW_2_ refers to the hard sphere, single and double square well systems respectively. The asterisk indicates that the system was run with 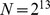 instead of 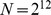. Note that in the SW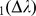 system, the singular values decay much slower suggesting that the system does not decompose as cleanly as the other variations.

We sampled 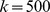 points in the parameter space for hard spheres HS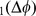 over 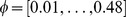 and 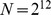. The six largest singular values in HS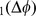 were exponentially separated from each other, as shown in Figure 1. Smaller singular values and their corresponding basis vectors contained only noise and did not improve fits. This strongly suggested that an accurate reconstruction of the original RDFs is possible using only a limited subset. To test for finite size effects, we repeated the HS simulation with twice as many particles, HS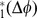, 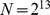. While the resolution of the basis vectors improved, this difference was negligible for all but the smallest vectors 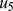 and 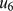.

**Figure 2:**
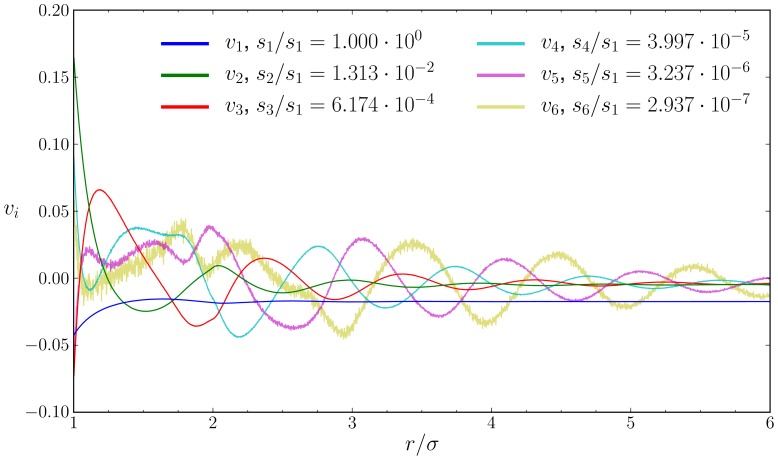
Five most dominant basis vectors 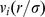 for the hard sphere system HS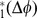. Results are identical, but less accurate, for the smaller 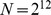 system. The vectors associated with the fourth and fifth singular values, 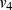 and 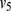, were at the sampling resolution and consequently had more noise.

**Figure 3:**
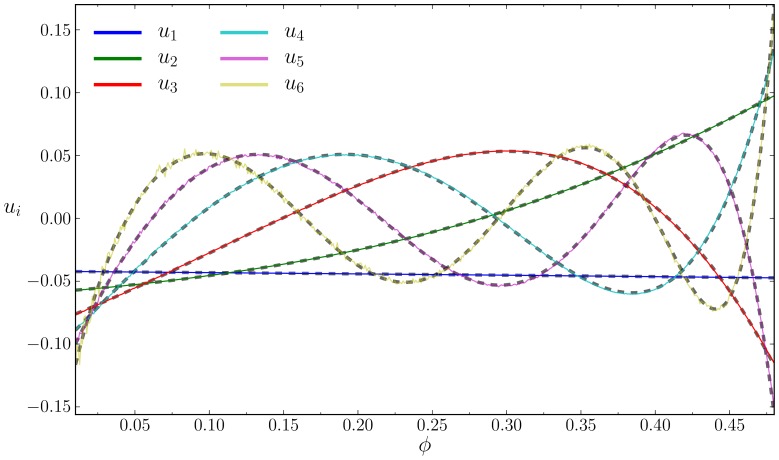
Five most dominant coefficient vectors 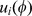 for the hard sphere system HS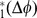. The polynomial fits to the vectors are shown as dashed lines.

In Figures 2 and 3 we plot both the basis and coefficient vectors. Somewhat surprisingly, the coefficient vector 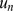 is extremely well described by a polynomial of degree 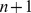, e.g. for the single parameterization of HS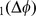 there exists a set of coefficients such that
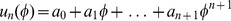
(4)with high accuracy. These polynomials contain 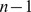 real roots and a single pair of complex conjugate roots. The polynomials fit so well to the coefficient vectors that they are indistinguishable for the dominant vectors and fit with little error for the smaller vectors. The log_10_ of the residuals from the polynomial fits, which we report for the more accurate HS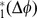, were −7.23, −5.00, −4.12, −3.59, −3.24, −2.56 for 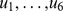 respectively. Due to the high quality of the fits, we only need to store the coefficients describing the polynomial to reproduce the coefficient vectors.

**Figure 4:**
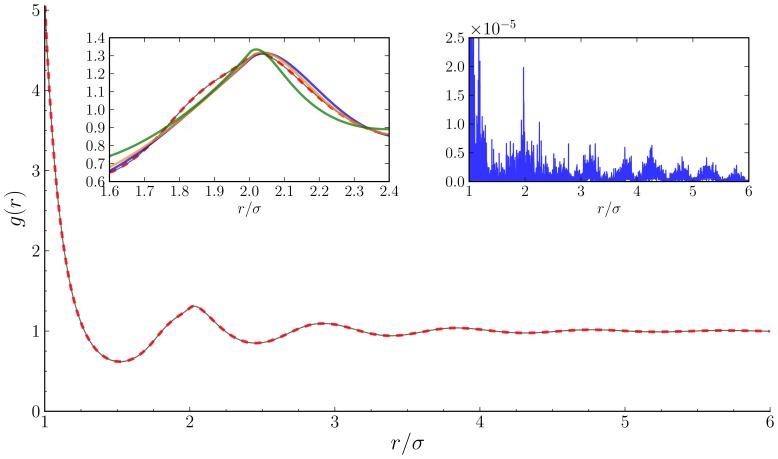
RDF for HS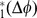 at *φ* = 0.46. The thin solid black line marks the computational value and the red dashed line is the six vector polynomial reconstruction from the SVD. Note that within the plotting resolution the lines are nearly identical. Left Inset: To illustrate the failure of the basic integrals closures, Percus Yevick (blue), Hypernetted chain (green), and Roger-Young (orange), we plot the predicted RDF calculated from [23] in the region of the second peak. Outside of this region, there is better agreement of the integral equations to the simulation results. Right Inset: Distance dependence on the residuals of the computed RDF 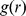 to the reconstructed RDF 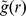, where the y-axis is 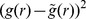.

In Figure 4 we show the measured RDF and the reduced polynomial reconstruction at a high packing fraction 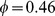. Within plotting accuracy, the two curves are identical. The squared difference between the curves shows excellent agreement, with the greatest discrepancy at the contact value. We compare our results with the basic predictions of classical fluid theory with three different closures, Percus Yevick (PY), Hypernetted chain (HNC), and the mixed form of Roger-Young (RY). For repulsive potentials, the HNC closure is is more appropriate for large interparticle distances and the PY closure works best at shorter distances [22]. The RY closure is a mixture of PY and HNC and typically outperforms both. The shape of the RDF near the second maxima for each of the closures calculated according to the method of Rogers [23] and is illustrated in the first inset. While all theories are fairly accurate, they fail to fully capture all the detail of the RDF. In contrast, the SVD reconstruction within the interpolated parameter space, does an excellent job in capturing the more problematic portion of the RDF.

### Square wells

**Figure 5:**
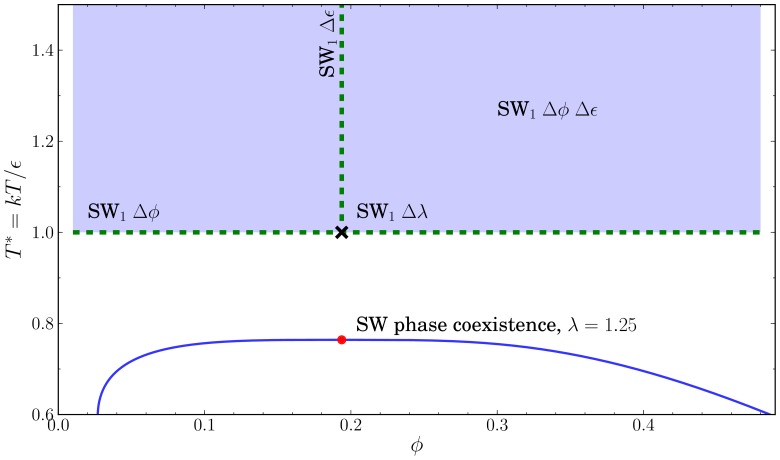
Parameter space of the SW, *λ* = 1.25 potential. The vertical and horizontal dashed lines indicate the variation for SW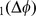 and SW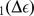 respectively. The cross indicates the point at which the well depth was varied SW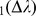, and the shaded region indicates the two parameter region explored SW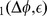. The vertical axis is plotted in 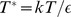 for comparison to known coexistence curves. As a reference, we show the liquid-vapor coexistence curve as a solid line, with a red dot at the critical point, 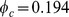, 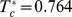, from Vega [24] and the coexistence curve determined via Gibbs-Duhem integration [29].

We explored a variety of parameters for the square well, varying packing fraction SW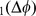, attractive strength SW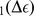, well length SW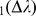, and a double well SW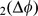. Like the HS system, we divided each parameter range into 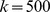 intervals with 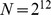, while holding the other parameters fixed. All values were above the liquid-vapor critical point and the freezing transition to ensure the system was in a single phase [24]. The specifics for each simulation along with an averaged error are summarized in Table 1. The SW system parameters are illustrated in Figure 5.

**Table 1:** Simulation parameters studied in this work.

	*φ*	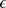	*λ*	*N*	avg. error
HS 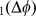 HS	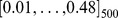	-	-	2^12^	.0064
HS 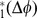 HS	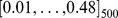	-	-	2^13^	.0046
SW 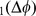 SW	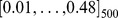	1.0	1.25	2^12^	.0093
SW 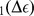 SW	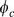	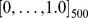	1.25	2^12^	.0050
SW 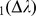 SW	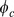	1.0	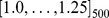	2^12^	.0350
SW 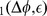 SW	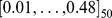	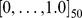	1.25	2^12^	.0086
SW 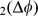 SW	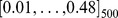	(−1.0, −1/2)	(1.125, 1.25)	2^12^	.0063

The subscript indicates the number of intervals the range was divided over. The SW parameter space is illustrated in Figure 5. 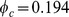 is the critical packing fraction for a SW at 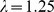. The double SW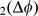 potential has two values for *λ* and 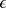 indicated in parentheses. The average error was the percentage difference between the measured *g*(*r*) and the SVD reconstructed 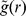 using the top five vectors, averaged over all system parameters: 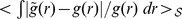. The domain of the integration extends from *σ* to 6*σ* since 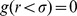 for the systems studied.

The singular values for the SW system, shown in Figure 1, indicate a sharp decomposition by *φ* and 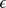. The spectrum of singular values for these systems level off within the first ten values indicating that the original signal can be reconstructed to high numerical precision. However, as a function of the well length *λ*, the singular values show a gentle slope with no dominant reduction, indicating that a reduced reconstruction is not feasible for this parameter. We conjecture that this is due to the location of the discontinuity changing in the potential. While the averaged error in Table 1 seems low, it is an order of magnitude larger than the other systems. The value of the RDF near the discontinuity 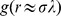 consistently undershoots the correct value with large oscillations, similar to that of a Gibbs phenomenon observed in the Fourier decomposition of a step function.

Since a SW is often a first-order approximation to a continuous potential, a double square well is the next logical step in the approximation. The system SW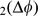 splits the well into two regions, giving three discontinuous points in the potential. Unlike the SW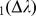 system, where the location of the discontinuity is changing, the double well potential was fixed as the other parameters varied. SW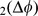 showed the sharp decomposition, suggesting that continuous potentials may be amenable to SVD. It is worth investigating whether other macroscopic potentials, such as Lennard-Jones, Yukawa, or even general anisotropic electrostatic models [25] can be decomposed in this manner. Very recently, the thermal stability of water and noble gases using a Lennard-Jones potential has been investigated [26], where similar results were obtained.

The basis vectors, which are given in the Supplementary Information, are similar to the HS except for the presence of discontinuities at the potential boundaries. The coefficient vectors also show the same general reduction to a polynomial of degree 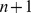. We also investigated the feasibility of a two parameter SVD in SW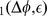. The singular values decayed more slowly than their one parameter counterparts but still much faster than SW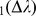. We found that the coefficient vectors, now a function of two variables, could be replaced by a bivariate polynomial
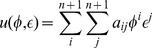
(5)


The static structure factor for an isotropic fluid can be calculated by a Fourier transform of the RDF, 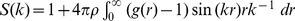. As a check for each reconstructed RDF, we have verified the physical requirement of positivity 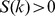 when at least five vectors were used.

## Discussion

While useful because of their compact representation, it is unclear if the exact polynomial representations of the coefficient vectors hold any special significance. Although they bear a resemblance to the radial Zernike polynomials [27], [28], all attempts at fitting proved unsuccessful. The existence of the pair of complex conjugate roots precluded the possibility that the coefficient vectors were formed directly from an orthogonal polynomial construction.

In theory, the basis functions themselves may yield qualitative insight, especially when the singular values are well separated. In the case of the RDF, it is possible that the basis functions are related to an expansion of the Ornstein-Zernike equation. For example, we find that the largest basis vector 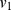 captures the scale of the potential, in that it approximates the contact value, any discontinuities, and captures the long range limit of 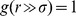. The less dominant basis functions seem to capture the variation in secondary maxima and minima as the parameters change. However, we are unable to ascertain any physical significance beyond these general qualitative observations. We also note that the reconstructed RDF's are only accurate within the interpolated parameter regions. While extrapolated values may be valid near the boundary of the parameter space, they are unlikely to give good predictions further way. The investigation of the polynomial representation of the coefficient vectors along with the connection of the basis vectors to the underlying liquid state theory also warrants additional study.

Preliminary work on both binary mixture and polydisperse hard sphere fluids show the same sharp separation of the singular values. These systems and other simple continuous potentials are being currently studied in more detail. For HS and SW systems we have shown that the reduced decomposition allows for the RDF to be reproduced with extremely high accuracy over all interpolated values of *φ* and 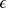.

### Supplementary Information

Source code to compute the radial distribution profiles for HS and SW can be found at https://github.com/thoppe/gr_svd.
